# Naso-oropharyngeal microbiome from breast cancer patients diagnosed with COVID-19

**DOI:** 10.3389/fmicb.2022.1074382

**Published:** 2023-01-11

**Authors:** Maria Carolina Viana, Gislaine Curty, Carolina Furtado, Bhavya Singh, Matthew L. Bendall, João P. B. Viola, Andreia Cristina de Melo, Marcelo A. Soares, Miguel A. M. Moreira

**Affiliations:** ^1^Tumor Genetics and Virology Program, Instituto Nacional de Câncer, Rio de Janeiro, Brazil; ^2^Division of Infectious Diseases, Department of Medicine, Weill Cornell Medicine, New York, NY, United States; ^3^Program of Immunology and Tumor Biology, Instituto Nacional de Câncer, Rio de Janeiro, Brazil; ^4^Division of Clinical Research and Technological Development, Instituto Nacional de Câncer, Rio de Janeiro, Brazil

**Keywords:** Breast cancer, COVID-19, SARS-CoV-2, microbiome, amplicon sequence variants, 16S-rDNA analysis

## Abstract

Due to immunosuppressive cancer therapies, cancer patients diagnosed with COVID-19 have a higher chance of developing severe symptoms and present a higher mortality rate in comparison to the general population. Here we show a comparative analysis of the microbiome from naso-oropharyngeal samples of breast cancer patients with respect to SARS-CoV-2 status and identified bacteria associated with symptom severity. Total DNA of naso-oropharyngeal swabs from 74 women with or without breast cancer, positive or negative for SARS-CoV-2 were PCR-amplified for 16S-rDNA V3 and V4 regions and submitted to massive parallel sequencing. Sequencing data were analyzed with QIIME2 and taxonomic identification was performed using the q2-feature-classifier QIIME2 plugin, the Greengenes Database, and amplicon sequence variants (ASV) analysis. A total of 486 different bacteria were identified. No difference was found in taxa diversity between sample groups. Cluster analysis did not group the samples concerning SARS-CoV-2 status, breast cancer diagnosis, or symptom severity. Three taxa (*Pseudomonas*, *Moraxella*, and *Klebsiella*,) showed to be overrepresented in women with breast cancer and positive for SARS-CoV-2 when compared to the other women groups, and five bacterial groups were associated with COVID-19 severity among breast cancer patients: *Staphylococcus*, *Staphylococcus epidermidis*, *Scardovia*, *Parasegitibacter luogiensis*, and *Thermomonas*. The presence of *Staphylococcus* in COVID-19 breast cancer patients may possibly be a consequence of nosocomial infection.

## 1. Introduction

The COVID-19 pandemic, caused by the severe acute respiratory syndrome coronavirus 2 (SARS-CoV-2), still represents an extreme concern given the peculiar and imprecise features of this illness. The vast majority of COVID-19 patients are asymptomatic or had mild respiratory symptoms (Huang et al., 2020; Wang et al., 2020), such as fever, dry cough, diarrhea and fatigue (WHO–World Health Organization, 2020a). However, patients with moderate symptoms (requiring medical hospitalization and non-invasive interventions) and severe cases (with O_2_ desaturation, dyspnea, septic shock, multiple organ dysfunction, and/or stroke), often causing death, were recorded, unveiling the severity of the disease (Huang et al., 2020; Wang et al., 2020). SARS-CoV-2 has infected more than 580 million people worldwide, causing more than 6.4 million deaths since January 2020 (Gandhi et al., 2020; Wang et al., 2020; Worldometer COVID-19 Data, 2022). Differently from the SARS-CoV induced disease, the upper respiratory tract is affected, even among pre-symptomatic patients, which may be associated with the high transmissibility of SARS-CoV-2 (Gandhi et al., 2020).

Although severe cases and deaths were reported in clinically healthy people, the presence of comorbidities was associated with the development of COVID-19 severe disease (CDC–Centers for Disease Control and Prevention, 2020a). Common comorbidities include diabetes, cardiovascular disease, hypertension, immunosuppression (solid organ transplantation, immunological deficiencies, HIV infection, and use of medications that compromise the immune system), and accumulated conditions in elderly persons (CDC–Centers for Disease Control and Prevention, 2020a; Sanyaolu et al., 2020). Additionally, male patients have higher odds of requiring intensive treatment care and also higher odds of death compared to women (Peckham et al., 2020). Cancer patients have a unique risk status due to a chronic immunosuppressive state consequent to the cytotoxic effects of anticancer treatments and use of corticosteroids (Addeo and Friedlaender, 2020; CDC–Centers for Disease Control and Prevention, 2020a; de Melo et al., 2020).

Several studies carried out in cancer patients with COVID-19 reported a higher chance of severe and critical symptoms, requiring invasive mechanical ventilation, admission to intensive care unit (ICU), and high mortality rates (Dai et al., 2020; Liang et al., 2020; Onder et al., 2020; Zhang et al., 2020). In the first study with a similar cohort conducted in Brazil, de Melo et al. (2020) evaluated the clinical characteristics of cancer patients with COVID-19 admitted to the Brazilian National Cancer Institute, reporting higher mortality rates among patients with lung (57.1%) and breast (52.5%) cancer.

Breast cancer is the most prevalent cancer worldwide (WHO–World Health Organization, 2020b, 2021; Sung et al., 2021) with >2 million new cases and > 684,000 deaths estimated for 2020. The COVID-19 and Cancer Consortium Registry database also showed breast cancer as the most frequent cancer among COVID-19 patients (Kuderer et al., 2020). On the other hand, Lasagna et al. (2021) hypothesized a relationship between the action of gut microbiota over estrogen metabolism in breast cancer patients and the susceptibility to COVID-19 in women diagnosed with breast cancer, considering the association between estrogen and COVID-19 severity and menopause as an independent risk factor for COVID-19 (Ding et al., 2021).

Recently, He et al. (2020) suggested that changes in the microbiota of COVID-19 patients may help to predict disease progression and the development of therapeutic strategies by understanding how changes in microbial communities affect viral SARS-CoV-2 infection. A study conducted focusing on the pulmonary microbial composition of patients with COVID-19 showed changes in the microbial composition in the bronchoalveolar lavage fluid when compared to clinically healthy controls (Shen et al., 2020): the microbiota was similar to that of patients with pneumonia, containing pathogenic bacterial strains or commensal bacteria found in the upper respiratory tract.

Changes in the microbiome can directly influence the homeostasis of the organism, modifying the capacity of immune response modulation, altering the physiology, and increasing the risk of viral infections and the development of diseases such as cancer (Gilbert et al., 2018; Curty et al., 2019; Rao et al., 2020). It can also affect cancer treatment by modifying gut and tumor microbiome (Sepich-Poore et al., 2021). Concerning breast cancer patients, changes in microbiome of breast tissue, milk ducts, distal gut, and the urinary tract were found in comparisons with health individuals (revised by Kovács et al., 2021). However, no significant differences were observed between oral samples (Wang et al., 2017), and to our knowledge no data have been reported for the nasopharyngeal region. Given that cancer patients are more susceptible to other infections and the association of COVID-19 with a higher mortality rate in these patients, it is relevant to analyze the microbiome of cancer patients diagnosed with SARS-CoV-2. In this work, we carried out a comparative analysis of the microbiome from naso-oropharyngeal regions from four sets of individuals: (i) breast cancer patients diagnosed with SARS-CoV-2; (ii) breast cancer patients without SARS-CoV-2 infection; (iii) individuals without breast cancer diagnosed with SARS-CoV-2; and (iv) individuals without breast cancer and not infected with SARS-CoV-2. Knowing the components of the microbiota and comparing those groups can shed light on the process triggered by viral infection in COVID-19 patients.

## 2. Materials and methods

### 2.1. Sample collection

Samples of 74 women were collected between April 26 and May 30, 2020 and analyzed in this work. This set included (Table 1): patients with breast cancer positive for SARS-CoV-2 (BC + CV+, *n* = 36); patients with breast cancer negative for SARS-CoV-2 (BC + CV–, *n* = 13); healthcare professionals, without cancer and positive for SARS-CoV-2 (BC–CV+, *n* = 20); and healthcare professionals without cancer and negative for SARS-CoV-2 (BC–CV–, *n* = 5). All breast cancer patients and healthcare professionals were from the Brazilian National Cancer Institute (Rio de Janeiro, RJ, Brazil), and were submitted to RT-PCR test due to COVID-19 symptoms or are breast cancer patients who had contact with COVID-19 cases, according to WHO interim guidance ((WHO-World Health Organization, 2020c). Differences with respect to age range were significant (*p* < 0.00001) between BC + CV+ *vs* BC-CV+ and between BC + CV+ *vs* BC-CV- (Mann Whitney *U*-test). All procedures were approved by the Institutional Research Ethics Committee and the Brazilian National Research Ethics Commission (CONEP; CAAE: 53398416.0.0000.5274) and carried out following the Good Clinical Practice guidelines, keeping participant identities confidential.

**Table 1:** Characterization of the women included in the study and their distribution over the study groups.

	BC+/CV+ (*n* = 36)	BC+/CV- (*n* = 13)	BC-/CV+ (*n* = 20)	BC-/CV- (*n* = 5)
Median age (min/max)^§^	60 yr. (41–86)	67 yr. (31–76)	37 yr. (27–63)*	44 yr. (43–54)**
Breast cancer stage				
1	1 (2.8%)	1 (7.6%)	NA	NA
2	3 (8.3%)	5 (39.5%)	NA	NA
3	10 (27.8%)	2 (15.4%)	NA	NA
4	22 (61.1%)	2 (15.4%)	NA	NA
Oxygen supplementation	31 (86.1%)	NA	NA	NA
ICU	3 (8.3%)	NA	NA	NA
Death by COVID-19	17 (47.2%)	NA	NA	NA

yr = age in years. Missing data: *five women; **one woman; NA, not applicable. ^§^Significant differences in age range between BC + CV+ vs BC-CV+ and BC + CV+ vs BC-CV- (*p* < 0.00001; Mann Whitney *U*-test).

Samples were collected by swabbing the nasal and oropharyngeal regions and mixed them in a single vial with PBS, according to the United States Centers for Disease Control and Prevention protocol (CDC–Centers for Disease Control and Prevention, 2020b), as described by de Melo et al. (2020). Nucleic acids were isolated from 200 μl of the naso-oropharyngeal swabs with the BioGene DNA/RNA viral isolation Kit (Bioclin, Belo Horizonte, Brazil) and stored at-80° C.

### 2.2. *16S* amplification and amplicon sequencing

For microbiota identification, a metagenomic approach was carried out through the high-performance sequencing of amplicons resulting from PCR of microbial 16S ribosomal DNA (16S-rDNA gene; Bragg and Tyson, 2014). PCR for 16S-rDNA was performed to amplify the V3 and V4 regions using the primers described by Herlemann et al. (2011), synthesized with adapters suited for construction of libraries, generating amplicons of approximately 460 bp. PCR was carried out in four sets performed simultaneously, and two negative controls per set were included. PCR were carried out in a final volume of 25 μl with 2.5 μl of DNA (5 ng/μl in 10 mM Tris pH 8.5); 1 μM of each primer; 0.3 mM dNTP; 1 U of the High-Performance GoTaq® G2 Hot Start Taq Polymerase (Promega Corp., Madison, WI), 5x PCR Buffer and 2.5 mM MgCl2. PCR products and negative controls were then submitted to electrophoresis in 1.5% agarose gels to verify amplicon expected size, and products were purified with the Illustra GFX PCR DNA and Gel Band Purification Kit (GE Healthcare, Chicago, IL), including five additional negative controls to monitor the presence of bacteria during these processes.

Purified products and 13 negative controls were quantified using Qubit™ fluorometer and 1X dsDNA HS (High-Sensitivity) Assay Kit (ThermoFisher Scientific, Waltham, MA). After quantification, 2.5–7 μl of each PCR product were used for indexing. For negative controls, indexing was carried out with 7 μl of the purified reaction. Indexing was carried out in 96-well plates in a final volume of 50 μl per reaction with 0.3 mM of each dNTP; 1 U of the High-Performance GoTaq® G2 Hot Start Taq Polymerase (Promega Corp.), 1x PCR Buffer and 2.5 mM MgCl2, 5 μl of Nextera XT Index Primer 1 (N7xx) and 5 μl of Nextera XT Index Primer 2 (S5xx; Nextera XT DNA Library Preparation Kit; Illumina, Inc., San Diego, CA). After indexing, samples were cleaned up using homogenized SPRI (Solid Phase Reversible Immobilization) paramagnetic AMPure XP PCR Purification beads (Thermo Fisher Scientific) according to the manufacturer’s protocol. Libraries were quantified by fluorometry using Qubit® Fluorometer and 1X dsDNA HS Assay and submitted to a 1.5% agarose gel electrophoresis to estimate amplicon length. Amplicon concentrations were estimated and the appropriate volume of each library was pooled in a single mix. Libraries were sequenced in a single pair-end run (300 × 300) in a MiSeq Next Generation Sequencer (Illumina) with the Nextera XT V3 kit (Illumina).

### 2.3. Data analysis

The BCL2FastQ2 conversion software (version 2.18, Illumina, Inc.) was used for demultiplexing and converting BCL files into FASTQ format. Reads were analyzed using FastQC (Babraham Bioinformatics, Cambridge, United Kingdom) for quality and quantity. The bacterial taxonomic analysis was conducted using QIIME2 (Bolyen et al., 2019). Reads were denoised, filtered and joined using the Divisive Amplicon Denoising Algorithm DADA2 (Callahan et al., 2016). The taxonomic classification was performed using the q2-feature-classifier QIIME2 plugin and the Greengenes Database, summarized in species taxonomic level (level 7; McDonald et al., 2012). Taxa were assigned using amplicon sequence variants (ASV) analysis.

The presence of a bacterial taxon in a sample was considered when the number of reads supporting this presence was higher than the number of reads for the ASV in the negative controls. Only the ASVs that fulfill this criterion were used in downstream analyzes described below. Rarefaction curves were built for each sample, including controls, to evaluate whether the number of reads can represent the bacterial diversity (Supplementary Figure S1). Differential abundance analysis was performed using DESeq2 (Love et al., 2014) and the significance of the differences found were evaluated using the Wald test; the bacteria with adjusted *p*-value <0.05 and absolute (log2FoldChange) > 1.0 were considered differentially abundant between groups of samples and the results presented using pheatmap (Kolde, 2019) and ggplot (Wickham, 2016) R packages. Unsupervised hierarchical clustering based on Bray–Curtis dissimilarity and average linkage was performed to define clusters according to abundance and taxa diversity of each sample. The alpha diversity between groups of samples was estimated using Shannon and Simpson diversity indexes and the differences found were compared using the Kruskal-Wallis (pairwise) test available in QIIME2 (Bolyen et al., 2019). Clustering, diversity analyses and plots were carried out using the R environment. To identify the taxa associated with the presence/absence of SARS-CoV-2 and of breast cancer, pairwise comparisons of bacteria relative abundance were carried out between the four groups. Bacteria over- and underrepresented between groups associated with COVID-19 diagnosis and/or breast cancer were listed and viewed in Edwards-Venn diagrams built using the interactive diagram viewer jvenn (Bardou et al., 2014).

Linear Discriminative Analysis (LDA) Effect Size (LEfSe; LDA-EfSe), which uses Kruskal-Wallis and estimates the effect size statistical metrics, was performed using the Galaxy environment to evidence potential bacterial markers to COVID severity in BC + CV+ patients. For this analysis, BC+CV+ patients were grouped into three categories according to COVID-19 severity: low (patients without respiratory symptoms), mild (patients with respiratory symptoms that received noninvasive oxygen support) and high severity (patients with severe respiratory symptoms that received mechanical ventilation). The association between bacterial taxa and disease severity was measured by estimating the odds ratio (OR) values and the 95% confidence intervals calculated using Haldane’s correction (adding 1 to each binary random variable to accommodate possible zero counts) considering the presence of a taxon for severity category.

## 3. Results

**Figure 1:**
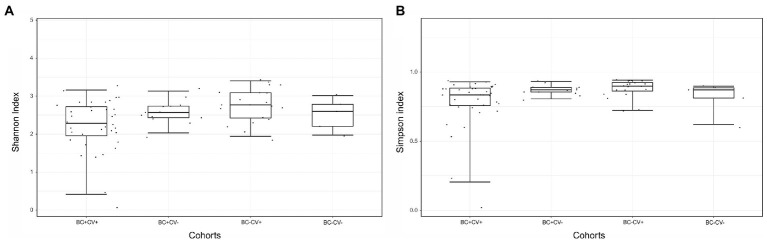
Comparison of alpha diversity estimates between the four groups of samples, based on **(A)** Shannon or **(B)** Simpson indexes. Comparisons were carried out with the Kruskal-Wallis (pairwise) test, no significant differences was found between groups.

NGS produced a total of 50,919,792 reads, including samples and controls (Supplementary Table S1). After quality filtering, a total of 37,627,622 reads with Q-scores >30 were kept for downstream analyses. Considering only the reads from libraries of the individuals included in the study, a mean of 324,260 reads per sample was produced. Considering only the reads of the 13 negative controls, a total of 77,287 reads were produced, with a mean of 5,945 reads per control. A total of 6,256,425 reads were attributed to bacterial 16S rDNA (Table 2), allowing the identification of 486 different bacteria; the number of distinct ASVs per group was: 192 for BC-CV-, 342 for BC-CV+, 232 for BC + CV-, and 318 for BC + CV+ (Table 2 and Supplementary Table S2). The analyses of bacterial diversity (Shannon and Simpson indexes) showed a lower median diversity for BC + CV+ patients. However, no significant difference was found between the four groups of patients concerning diversity indexes (Figure 1).

**Table 2:** Number of reads with 16S sequences (V3 and V4 regions) and number of taxa identified per group.

	BC+/CV+ (*n* = 36)	BC+/CV- (*n* = 13)	BC-/CV+ (*n* = 20)	BC-/CV-(*n* = 5)
Total number of reads	3,330,724	1,286,572	1,253,356	385,773
Mean	140,5	112	114	140
Min.	2	1	1	2
Max.	448,306	185,785	46,991	55,879
Total number of taxa	318	232	342	192

**Figure 2:**
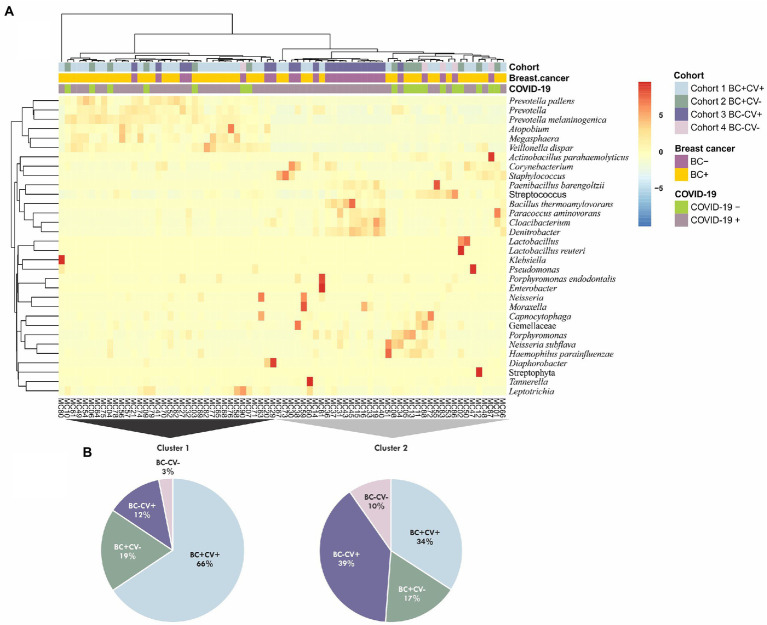
**(A)** Cluster analysis considering all taxa identified in the four groups of samples (CV + BC+, CV-BC+, CV + BC-, and CV-BC-). The analysis was performed using unsupervised hierarchical inferences based on Bray–Curtis dissimilarity and average linkage considering the abundance and taxa diversity of each sample; the heatmap shows the taxa with relative abundance ≥1%. **(B)** Pie charts showing the contribution of the different groups of samples for the two major clusters (Cluster 1 and Cluster 2), colors are respective to each cohort as presented in **A**.

To assess a possible association between relative abundance of the bacteria identified and breast cancer and SARS-CoV-2, an unsupervised cluster analysis was performed considering all ASVs identified. Two major clusters were retrieved; however, these clusters did not group all samples based on SARS-CoV-2 status or breast cancer diagnosis. Samples of the four groups of individuals were intermingled in different clusters (Figure 2A). Samples from BC-CV+ patients were the most frequent in Cluster 2 and BC + CV+ in Cluster 1 (Figure 2B); all BC-CV-samples, but one, were grouped in Cluster 2. The taxa with highest relative abundance were *Staphylococcus, Streptococcus, Corynebacterium, Cloacibacterium, Denitrobacter*, and *Prevotella* in Cluster 1; and *Prevotella melaninogenica*, *Veillonella dispar*, *Streptococcus*, *Prevotella*, *Neisseria subflava*, and *Leptotrichia* in Cluster 2 (Supplementary Table S3).

**Figure 3:**
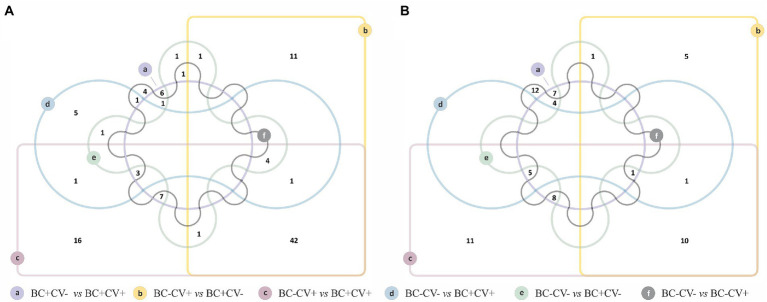
Edwards-Venn diagrams showing the number of bacterial taxa with significant differential relative abundance between each pairwise comparison of sample groups (CV + BC+, CV-BC+, CV + BC-, and CV-BC-). **(A)** Number of taxa overrepresented and shared between each comparison; **(B)** number of taxa underrepresented and shared between each comparison.

Of the six comparisons carried out to identify the taxa with differential relative abundance associated with SARS-CoV-2 status and breast cancer, the comparison between BC-CV+ vs. BC + CV+ showed the largest number of ASVs (*n* = 111) with significant differences in relative abundance. On the other hand, BC-CV-vs. BC + CV-presented the lowest number of bacteria with significant differences in relative abundance (*n* = 10). Edwards-Venn diagrams summarizing the number of over- and underrepresented taxa for COVID-19 diagnosis and/or breast cancer are shown in Figure 3, and taxa are listed in Supplementary Table S4. Comparisons carried out between BC + CV+ women with respect to the remaining women groups (BC + CV, BC-CV, and BC-CV-), showed that eight ASV recurrently presented significant differential relative abundance, being five overrepresented (*Pseudomonas*, *Moraxella*, *Klebsiella*, *Schwartzia*, *Lactobacillus salivariuspresen*) and three underrepresented (*Rhodobacter*, *Actinobacillus parahaemolyticus*, *Diaphorobacter*) in BC + CV+. *Pseudomonas*, *Moraxella*, and *Klebsiella* were found to be the three most overrepresented ASV in BC + CV+ when compared with the other women groups (Supplementary Table S4).

**Figure 4:**
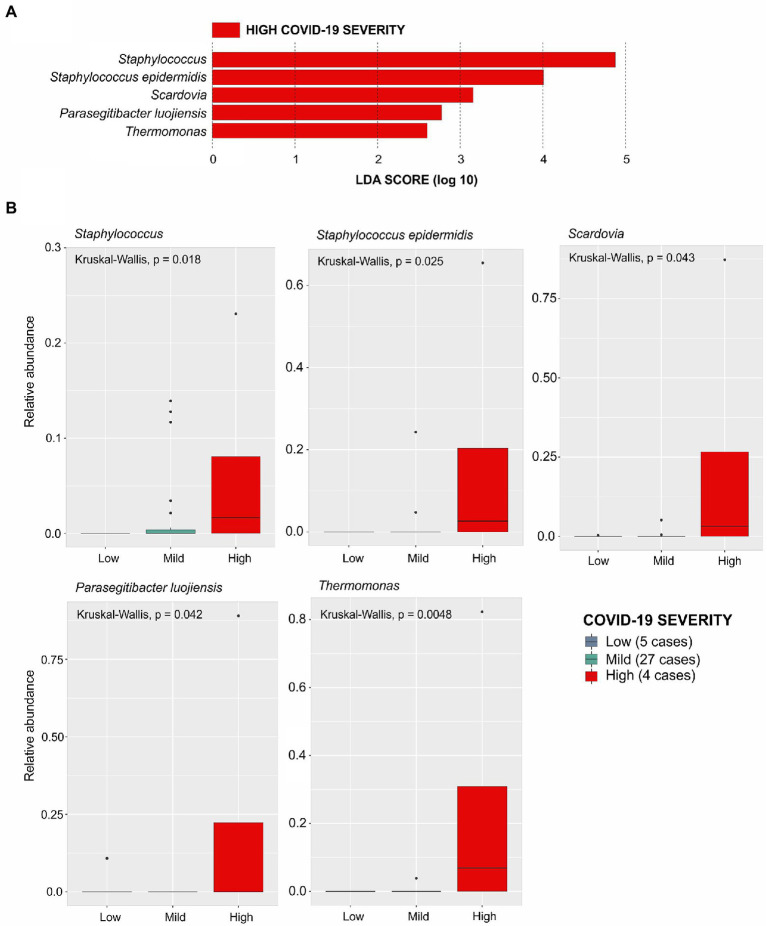
Linear Discriminative Analysis - Effect Size (LDA-EfSe) to identify bacterial taxa associated with COVID severity in BC + CV+ patients: **(A)** Bacterial taxa associated with disease severity; **(B)** Histograms showing the comparison of the relative abundance of each bacterial taxa between the BC + CV+ patients discriminated with respect to COVID severity (low, mild, or high). Comparisons were carried out using the Kruskal-Wallis test, and the *p*-values are presented for each taxon.

To identify the taxa associated with COVID-19 severity among patients with breast cancer (BC + CV+), we performed the LEfse analysis considering patients grouped according to disease severity: with low severity (*n* = 5), with mild severity (*n* = 27), and with high severity (*n* = 4; see Material and Methods). This analysis evidenced five bacteria associated with high severity: *Staphylococcus*, *Staphylococcus epidermids*, *Scardovia*, *Parasegitibacter luogiensis*, and *Thermomonas* (Figures 4A,B). The *Staphylococcus* genus was strongly associated with COVID-19 high severity, with patients presenting the higher relative abundance of this taxon in comparison with the other patient groups. Four of those five taxa were also listed among those with significant differences in relative abundance in the comparisons between the four groups of samples: *Staphylococcus epidermids*, *Scardovia*, *Parasegitibacter luogiensis*, and *Thermomonas* for BC-CV+ vs. BC + CV+; *Staphylococcus epidermids*, *Parasegitibacter luogiensis*, and *Thermomonas* for BC-CV+ vs. BC + CV-; *Parasegitibacter luogiensis* and *Thermomonas* for BC-CV+ vs. BC-CV-; and *Scardovia* for BC-CV+ vs. BC + CV+. None of those five taxa was found to be differentially abundant between BC + CV-*vs* BC + CV+, and *Staphylococcus* was not found to be differentially abundant between the four groups.

Considering the presence of each of these five taxa and disease severity (Supplementary Table S5), an association was found only for *Staphylococcus*, when comparing patients with low *vs*. high severity, indicating that the abundance of this taxon increases with severity (OR = 30; CI 95% = 1.5–611.8).

## 4. Discussion

Given that cancer patients are more susceptible to viral infections as a result of immunosuppressive anticancer treatments, and the association of COVID-19 with requirement of mechanical ventilation, ICU stay, hospitalization, and a high mortality among patients submitted to recent treatment (Chavez-MacGregor et al., 2022), it is relevant unrevealing the microbiome of cancer in patients diagnosed with SARS-CoV-2. In this study, we analyzed and compared the microbiome of patients with breast cancer with respect to the diagnosis of SARS-CoV-2 and disease severity and with those patients without cancer.

Although differences in the relative abundance between the four groups of samples were found for specific taxa, the Shannon and Simpson diversity indexes were not significantly distinct. Interestingly, the pairwise comparisons between the four groups showed that women with breast cancer and positive for COVID-19 (BC + CV+) presented the highest difference in relative abundance for *Pseudomonas*, *Moraxella*, and *Klebsiella*. Among these genera, *Klebsiella* was previously associated with COVID-19 severity (Ma et al., 2021). On the other hand, the higher relative abundance of *Pseudomonas* and *Moraxella* were also reported in individual positive for SARS-CoV-2 by Gupta et al. (2022). In this work we found that both genera were those with highest differences in relative abundance between BC + CV+ women compared with other women groups. This suggest that changes in upper respiratory tract caused by viral infection are distinct in women diagnosed with breast cancer, promoting the proliferation of these bacteria, considered opportunistic pathogens.

Considering COVID-19 severity (requirement of supplemental oxygen or mechanical ventilation) in breast cancer patients, we found a direct relationship with relative abundance of five taxa: *Staphylococcus*, *Staphylococcus epidermids*, *Scardovia*, *Parasegitibacter luogiensis* and *Thermomonas*. With respect to *Staphylococcus*, such association was likely the result of nosocomial infection by *Staphylococcus*, since 10/13 (77%) breast cancer patients positive for SARS-Cov-2 that required oxygen or mechanical ventilation were already hospitalized at the time of sample collection for viral diagnosis. Regarding *Scardovia*, Rueca et al. (2021) also report the presence of this genus exclusively in COVID-19 patients admitted to intensive care units. For *Parasegitibacter luogiensis* and *Thermomonas*, the higher abundance is likely resulted from opportunistic infections, as *Parasegitibacter* is associated with soil and skin microbiome (Manus et al., 2020), and *Thermomonas* is common in air pollution (Chen et al., 2022).

This study has some limitations as all samples consist of a mix of nasal and oropharyngeal regions obtained by swabbing. Therefore, it is not possible to link the presence of each bacterium with a specific human anatomical topographic region. Other limitation is the potential confounding effect of the differences in age between breast cancer patients positive for COVID-19 and women without cancer. The inclusion of samples collected in a longer period of time, and matched by age, could minimize this possibility, however, it would allow the influence of distinct SARS-CoV-2 variants. The findings presented here are restricted to the beginning of COVID-19 pandemic in Brazil, and cannot be extended to other periods of the COVID-19 pandemic when distinct SARS-CoV-2 variants of concern predominated.

In conclusion, our data did not provide evidence of a discreet change in naso-oropharyngeal microbiome diversity in breast cancer patients with COVID-19 when compared with SARS-CoV-2 negative breast cancer patients or with individuals without cancer. On the other hand, we found that the presence of SARS-CoV-2 was associated with an increase in the relative abundance of specific bacteria in breast cancer patients when compared with other groups. Moreover, we found that COVID-19 severity is associated with a higher relative abundance of five bacterial taxa, being the presence of *Staphylococcus* associated with severe respiratory symptoms and patients requiring oxygen support by mechanical ventilation, likely reflecting hospital-acquired infections.

## Data availability statement

The information was provided as suggested and the data is available: http://www.ncbi.nlm.nih.gov/bioproject/repository accession number PRJNA906512.

## Ethics statement

This study involving human participants were reviewed and approved by Institutional Research Ethics Committee and the Brazilian National Research Ethics Commission (CONEP; CAAE: 53398416.0.0000.5274). Written informed consent for participation was not required for this study in accordance with the national legislation and the institutional requirements.

## Author contributions

MCV and MAMM conceived the study design. GC carried out the bioinformatic analyses. MCV carried out laboratory procedure for library preparation. CF carried out sequencing procedures. BS and MLB contributed with bioinformatic analyses and data interpretation. JPBV coordinated the INCA COVID-19 task force. ACM was responsible for clinical data collection. MAS coordinated the COVID-19 diagnosis, sample storage, and sample availability. MCV, GC, MAS, and MAMM wrote the manuscript. All authors approved the final manuscript.

## Funding

This work was supported by grants from Fundação Carlos Chagas Filho de Amparo à Pesquisa do Estado do Rio de Janeiro, Brazil (FAPERJ) grants # 202.640/2019, 211.562/2019, 010.000162/2020 (to JPBV) and 200.928/2021 (to MAMM); Conselho Nacional de Desenvolvimento Científico e Tecnológico, Brazil (CNPQ) grants # 307042/2017–0 (to JPBV), 305765/2015–9 (to MAS) and 304339/2018–0 (to MAMM); by the Ministry of Health (MAS), Brazil; and Instituto Nacional de Câncer, Brazil (INCA) (intramural grants). MCV and GC were supported by INCA/Ministry of Health-Brazil fellowships.

## Conflict of interest

The authors declare that the research was conducted in the absence of any commercial or financial relationships that could be construed as a potential conflict of interest.

## Publisher’s note

All claims expressed in this article are solely those of the authors and do not necessarily represent those of their affiliated organizations, or those of the publisher, the editors and the reviewers. Any product that may be evaluated in this article, or claim that may be made by its manufacturer, is not guaranteed or endorsed by the publisher.
