# Supplementary figures and images for: Naso-oropharyngeal microbiome from breast cancer patients diagnosed with COVID-19
Source: Front Microbiol. 2023 Jan 11;13:1074382. doi: 10.3389/fmicb.2022.1074382 (PMC9874304; doi:10.3389/fmicb.2022.1074382)

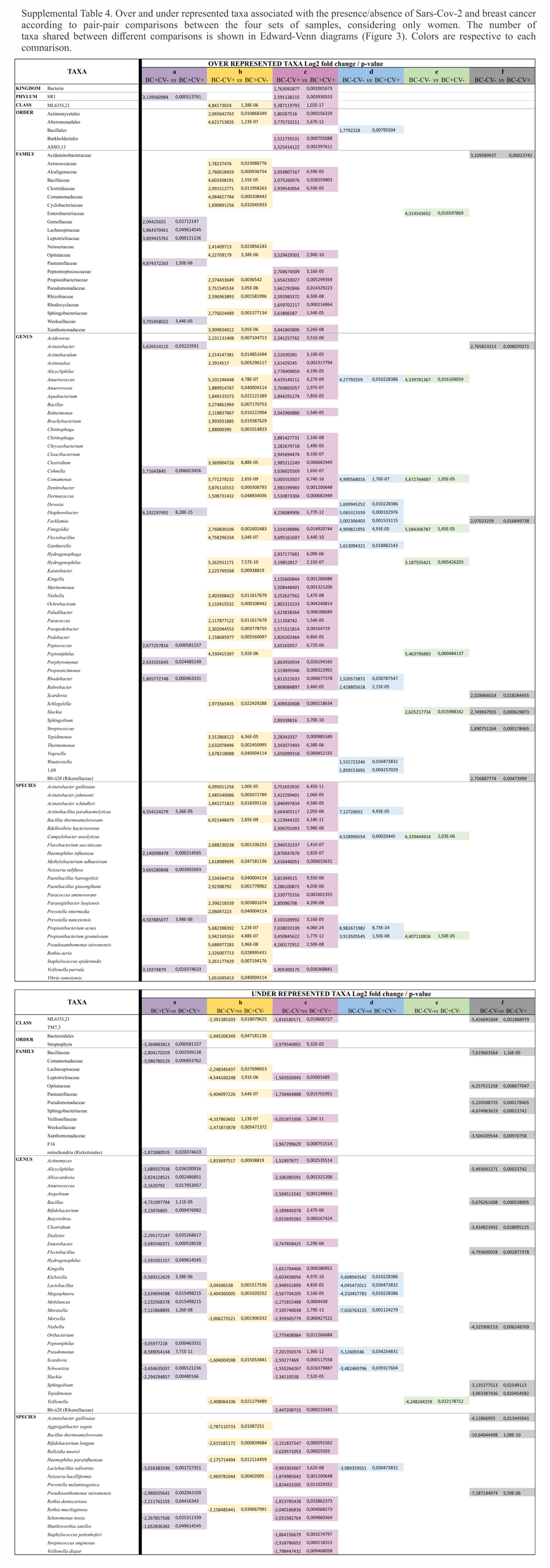

Supplement: Supplementary file 1 [file Image_1.JPEG]

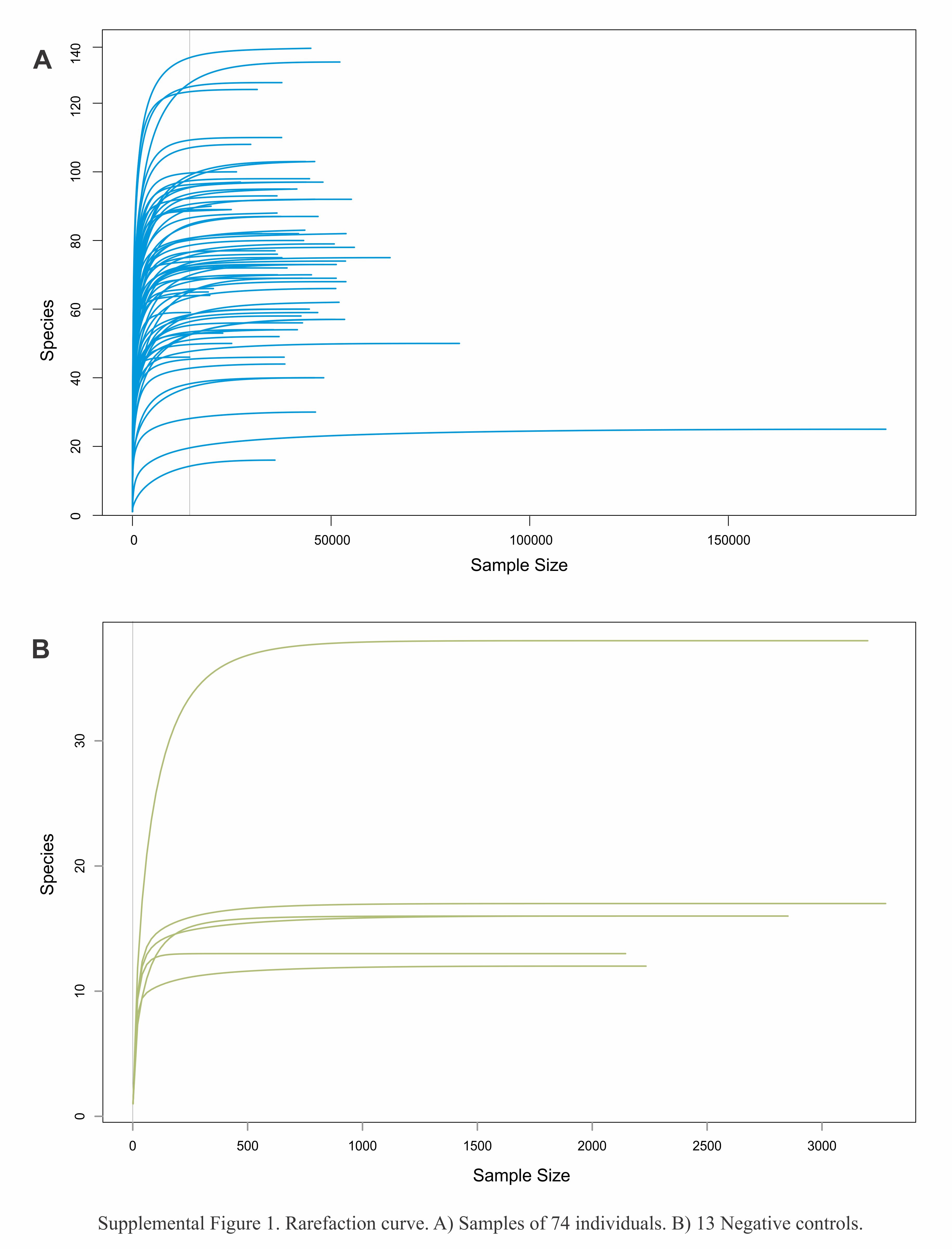

Supplement: Supplementary file 2 [file Image_2.JPEG]
