# Supplementary material for: Naso-oropharyngeal microbiome from breast cancer patients diagnosed with COVID-19
Source: Front Microbiol. 2023 Jan 11;13:1074382. doi: 10.3389/fmicb.2022.1074382 (PMC9874304; doi:10.3389/fmicb.2022.1074382)
Supplement: Supplementary file 3 [file Table_1.DOCX]

Supplemental Table 1. MiSeq results metrics.

| Sampling | Quality filtering reads Q-scores >30 | Mean per sample | Median of reads (min - max) |
| --- | --- | --- | --- |
| Total samples (N=74) | 6,256,425 | 126,620 | 245,224  (70,164 to 525,266) |
| Negative controls (N=13) | 77,287 | 5,945 | 2,671  (113 to 13,282) |
