# Supplementary material for: Naso-oropharyngeal microbiome from breast cancer patients diagnosed with COVID-19
Source: Front Microbiol. 2023 Jan 11;13:1074382. doi: 10.3389/fmicb.2022.1074382 (PMC9874304; doi:10.3389/fmicb.2022.1074382)
Supplement: Supplementary file 6 [file Table_5.DOCX]

Supplemental Table 5. Odds ratio (OR) for the presence of the bacterial taxa identified by LDA-Efse analysis; comparisons between low severity and mild (MS) or high severity (HS) COVID-19 cases.

| Taxa | Status | Low severity (LS) vs Mild severity (MS) | | | Low severity (LS) vs High severity (HS) | | | |
| --- | --- | --- | --- | --- | --- | --- | --- | --- |
|  |  | LS (%) | MS (%) | OR (CI95) ^1^ | LS (%) | HS (%) | | OR (CI95) ^1^ |
| *Thermomonas* | presence | 0/5 (0) | 1/27 (3.7) | 0.4 (0 - 5.7) | 0/5 (0) | 2/4 (50) | 6 (0.42 - 85.3) | |
|  | absence | 5/5 (100) | 26/27 (96.3) |  | 5/5 (100) | 2/4 (50) |  |  |
| *Staphylococcus* | presence | 0/5 (0) | 9/27 (33.3) | 3.16 (0.3 - 29.9) | 0/5 (0) | 4/4 (100) | 30 (1.5 - 611.8)*** | |
|  | absence | 5/5 (100) | 18/27 (66.6) |  | 5/5 (100) | 0/4 (0) |  |  |
| *Scardovia* | presence | 1/5 (20) | 2/27 (7.4) | 0.32 (0 - 4.4) | 1/5 (20) | 2/4 (50) | 4 (0.2 - 75.7) | |
|  | absence | 4/5 (80) | 25/27 (92.6) |  | 4/5 (80) | 2/4 (50) |  |  |
| *Parasegitibacter luojiensis* | presence | 1/5 (20) | 0/27 (0) | 0.08 (0 - 1.18) | 1/5 (20) | 1/4 (25) | 1.3 (0.1 - 31.1) | |
|  | absence | 4/5 (80) | 27/27 (100) |  | 4/5 (80) | 3/4 (75) |  |  |
| *Staphylococcus epidermidis* | presence | 0/5 (0) | 2/27 (7.4) | 0.6 (0 - 7.87) | 0/5 (0) | 2/4 (50) | 6 (0.42 - 85.3) | |
|  | absence | 5/5 (100) | 25/27 (92.6) |  | 5/5 (100) | 2/4 (50) |  |  |

^1^ Odds ratios were calculated with Haldane’s modification, which adds 1 to all cells to accommodate possible zero counts. * Significant OR (p< 0.05), confidence interval higher than 1.
